# Consistent B Cell Receptor Immunoglobulin Features Between Siblings in Familial Chronic Lymphocytic Leukemia

**DOI:** 10.3389/fonc.2021.740083

**Published:** 2021-08-26

**Authors:** P. Martijn Kolijn, Alice F. Muggen, Viktor Ljungström, Andreas Agathangelidis, Ingrid L. M. Wolvers-Tettero, H. Berna Beverloo, Karol Pál, Paul J. Hengeveld, Nikos Darzentas, Rudi W. Hendriks, Jacques J. M. van Dongen, Richard Rosenquist, Anton W. Langerak

**Affiliations:** ^1^Laboratory Medical Immunology, Department of Immunology, Erasmus MC, University Medical Center, Rotterdam, Netherlands; ^2^Department of Immunology, Genetics and Pathology, Science for Life Laboratory, Uppsala University, Uppsala, Sweden; ^3^Department of Molecular Medicine and Surgery, Karolinska Institutet, Stockholm, Sweden; ^4^Department of Clinical Genetics, Karolinska University Laboratory, Karolinska University Hospital, Solna, Sweden; ^5^Institute of Applied Biosciences, Centre for Research and Technology Hellas, Thessaloniki, Greece; ^6^Department of Biology, School of Science, National and Kapodistrian University of Athens, Athens, Greece; ^7^Department of Clinical Genetics, Erasmus MC, University Medical Center, Rotterdam, Netherlands; ^8^CEITEC – Central European Institute of Technology, Masaryk University, Brno, Czechia; ^9^Department of Hematology, University Hospital Schleswig-Holstein, Kiel, Germany; ^10^Department of Pulmonary Medicine, Erasmus MC, University Medical Center, Rotterdam, Netherlands; ^11^Department of Immunology, LUMC, Leiden, Netherlands

**Keywords:** CLL (Chronic Lymphocytic Leukemia), Familial CLL, BCR stereotypy, IGLV3-21 R110, CLL development

## Abstract

Key processes in the onset and evolution of chronic lymphocytic leukemia (CLL) are thought to include chronic (antigenic) activation of mature B cells through the B cell receptor (BcR), signals from the microenvironment, and acquisition of genetic alterations. Here we describe three families in which two or more siblings were affected by CLL. We investigated whether there are immunogenetic similarities in the leukemia-specific immunoglobulin heavy (IGH) and light (IGL/IGK) chain gene rearrangements of the siblings in each family. Furthermore, we performed array analysis to study if similarities in CLL-associated chromosomal aberrations are present within each family and screened for somatic mutations using paired tumor/normal whole-genome sequencing (WGS). In two families a consistent IGHV gene mutational status (one IGHV-unmutated, one IGHV-mutated) was observed. Intriguingly, the third family with four affected siblings was characterized by usage of the lambda IGLV3-21 gene, with the hallmark R110 mutation of the recently described clinically aggressive IGLV3-21^R110^ subset. In this family, the CLL-specific rearrangements in two siblings could be assigned to either stereotyped subset #2 or the immunogenetically related subset #169, both of which belong to the broader IGLV3-21^R110^ subgroup. Consistent patterns of cytogenetic aberrations were encountered in all three families. Furthermore, the CLL clones carried somatic mutations previously associated with IGHV mutational status, cytogenetic aberrations and stereotyped subsets, respectively. From these findings, we conclude that similarities in immunogenetic characteristics in familial CLL, in combination with genetic aberrations acquired, point towards shared underlying mechanisms behind CLL development within each family.

## Introduction

Chronic lymphocytic leukemia (CLL) is the most common leukemia in Western countries (1). Sex and age are important risk factors for CLL, with a two-fold increased risk of developing CLL for men compared to women and a median age at CLL diagnosis of around 70 years (2). Although no single genetic lesion drives CLL, a range of recurrent cytogenetic aberrations and somatic mutations have been identified in CLL (2–4).

Cytogenetic aberrations are common in CLL, with around 80% of CLL patients carrying at least one of the four common chromosomal alterations, i.e. del(13q), del(11q), del(17p) and trisomy 12 (2, 5). Of these four alterations, del(13q) is the most frequent and, as a sole aberration, is associated with indolent disease (6). Del(11q) and del(17p) are associated with an unfavorable prognosis, through loss of function of the *ATM* and *TP53* gene, respectively (3, 7–9). Lastly, trisomy 12 is associated with an intermediate prognosis (10, 11). Several key whole-exome sequencing (WES) and whole-genome sequencing (WGS) studies have revealed over 50 recurrently mutated genes (4, 12–15). However, the majority of these putative CLL driver mutations are present at low frequency (<5% of cases), with only a handful of more common mutations in genes such as *TP53*, *ATM*, *SF3B1, NOTCH1* and *BIRC3* (4, 12).

Another important facet of risk stratification of patients with CLL is the somatic hypermutation (SHM) status of the B cell receptor (BcR) immunoglobulin heavy variable (IGHV) gene (16). CLL patients with a mutated IGHV-gene (M-CLL), i.e. showing lower than 98% IGHV gene similarity to its closest germline counterpart, generally have a more indolent disease course than CLL patients with an unmutated IGHV gene with a germline identity equal to or above 98% (U-CLL) (2). Furthermore, stereotyped or (quasi)identical BcR IGs are observed in more than 40% of CLL patients (16). Patients with shared BcR IG motifs can be assigned to distinct stereotyped subsets associated with particular presentation and outcomes (17, 18). One of the stereotyped subsets with the worst clinical outcome is subset #2 (IGHV3-21/IGLV3-21), which displays a mixed IGHV mutation status and an enrichment of *SF3B1* mutations (17–19). An important new subset is the clinically aggressive IGLV3-21^R110^ subset, which also includes subset #2, that is characterized by shared usage of the lambda IGLV3-21*01 or *04 allele, along with a hallmark substitution of Gly to Arg at amino acid position 110 at the very end of the IGLJ gene (20, 21). The IGLV3-21*01 and *04 alleles encode a Lys at position 16 and two Asp residues at position 50 and 52 in the CDR2 region of the light chain variable region (VL), which interact with the R110 light chain residue, resulting in constitutive autostimulation of the BcR, putatively contributing to CLL pathogenesis (20).

Although the aforementioned genetic features mostly occur sporadically, evidence exists for germline predisposition for CLL (17, 22). The incidence of CLL varies geographically, with highest incidence among individuals with European ancestry (23). This hereditary element of CLL is also reflected in familial predisposition, as relatives of CLL patients have an increased risk of developing CLL as well as other B-cell malignancies (24). Furthermore, monoclonal B-cell lymphocytosis (MBL), the asymptomatic pre-stage to CLL, is more often seen in first-degree relatives of CLL patients and is particularly common among healthy relatives of patients with high-risk familial CLL (i.e. families with two or more relatives with CLL) with a prevalence of around 15% among individuals older than 40 years (23, 25). Genome-wide association studies (GWAS) have captured part of this familial predisposition by screening for single nucleotide polymorphisms (SNP) associated with familial CLL, yielding low-risk SNPs distributed over nearly 30 loci (22, 26–33).

In this context, through a combination of immunogenetic, SNP-array and WGS analysis, we here aimed to gain insight into the contribution of BcR composition, cytogenetic aberrations and CLL driver mutations to familial CLL occurrence by studying three families with multiple siblings diagnosed with CLL.

## Materials and Methods

### Samples

**Figure 1:**
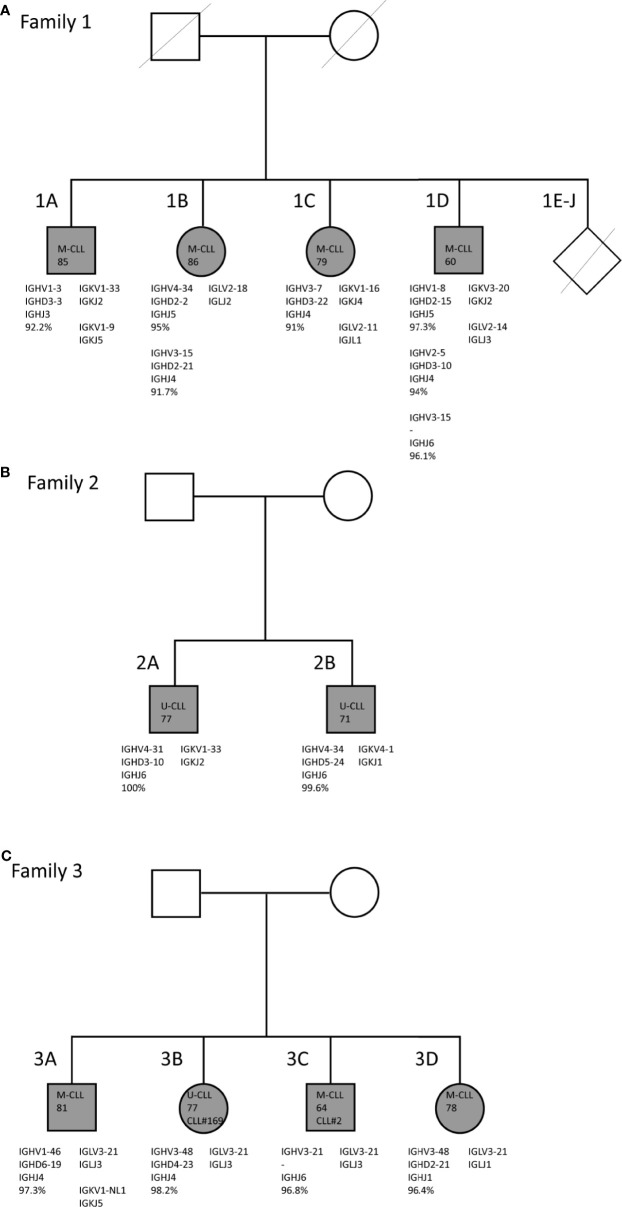
Family trees and BcR IG characteristics of familial CLL cases. **(A)** Family 1 consists of two brothers and two sisters who carry mutated IGHV genes. **(B)** Family 2 consists of two brothers who both carry unmutated IGHV genes. **(C)** Family 3 consists of two brothers and two sisters. In all four siblings, the CLL clone utilizes the IGLV3-21*04 gene with the characteristic R110 mutation and the K16 and YDSD motifs. Additionally, siblings 3B and 3C express similar IGHV genes, i.e. IGHV3-21 and IGHV3-48, and belong to stereotyped subsets #2 and #169, respectively.

Peripheral blood was obtained from ten CLL patients from three families (**Figure 1**). Informed consent was provided in accordance with the declaration of Helsinki and the study was approved by the hospital medical ethics committee (METC2015-741). Familial connection was confirmed through STR analysis. Peripheral blood mononuclear cells (PBMCs) were isolated by Ficoll Paque (GE Healthcare, Little Chalfont, UK) gradient centrifugation. CLL cells and T lymphocytes were sorted from PBMCs using a FACSAria cell sorter (BD Biosciences, San Jose, CA, USA). Immediately after sorting, cells were lysed in RLT+ buffer (Qiagen, Valencia, CA, USA) complemented with β-mercapto-ethanol and stored at -80°C until further processing. DNA and RNA was isolated with the DNA/RNA/miRNA easy kit (Qiagen) according to the manufacturer’s protocol. In the event that DNA was isolated from total PBMC, spin-column kits and the QIAcube platform (Qiagen) were used. cDNA was synthesized using the SuperScript™ III First-Strand Synthesis System (Thermo Fisher Scientific, Waltham, MA, USA), according to manufacturer’s instructions.

### IG Gene Rearrangement Analysis

Immunoglobulin heavy (IGH) and IG kappa/lambda (IGK/IGL) gene rearrangements were amplified from 100 ng gDNA isolated from the total PBMC fraction with multiplex PCR utilizing the BIOMED-2 IGH primers and IG light chain consensus primers, following ERIC guidelines (34, 35). Clonal PCR products were separated by heteroduplex gel electrophoresis and were purified by gel extraction. Rearrangements were determined through Sanger sequencing on an ABI 3130xl instrument (ThermoFisher Scientific, Waltham, MA, USA). Sequencing results were analyzed using the IMGT/V-QUEST tool on the IMGT website (www.imgt.org, version 3.3.1). Stereotyped subsets were defined by the following parameters: (1) usage of IGHV genes from the same phylogenetic clan, (2) a minimum of 50% amino acid identity and 70% similarity within the heavy chain CDR3, (3) identical heavy chain CDR3 length and, (4) identical offset of the shared amino acid pattern (28). The IGLV3-21^R110^ mutation was confirmed using IGLV3-21 primers on cDNA for 3 out of 4 members of family 3. As no RNA was available for sibling 3C, the R110 mutation was instead confirmed based on the WGS results analyzed by an extension of the ARResT/Interrogate immunoprofiler for the analysis of IG/TR rearrangements in non-amplicon sequencing data such as from WGS, WES and RNA-seq (36, 37).

### SNP Array Analysis

Two hundred fifty ng of genomic DNA was used for single nucleotide polymorphism (SNP) array analysis on the Illumina Human OmniExpress Beadchip (Illumina, San Diego, CA, USA). Data were analyzed with Beadstudio software (Illumina). The log R ratio and B allele frequency data were analyzed using Nexus Copy Number (Nexus BioDiscovery, El Segundo, CA, USA). The results were compared with a database of known copy-number variations (Department of Clinical Genetics, Erasmus MC, Rotterdam, The Netherlands) and a public copy-number variations dataset containing approximately 3500 healthy controls (dataset of genomic variants). The affected locations detected were analyzed in Ensembl Genome Browser 95 (www.ensembl.org) and screened for loci previously linked to CLL in GWAS studies. The used SNP array contained more than 700K probes, and the genome was analyzed with an average resolution of 150 kb, or smaller when it contained at least 10 consecutive probes.

### Whole-Genome Sequencing

One hundred ng of genomic DNA was used for construction of WGS libraries using the TruSeq Nano Kit (Illumina Inc.) and sequenced in paired-end mode (2x150bp) on the Illumina HiSeqX Ten system (Illumina Inc.) with 30× target coverage. The bcl files were converted to FASTQ using bcl2fastq and subsequently processed using Piper, a pipeline built on top of GATK queue. Reads from each library were aligned to the Grch37 reference genome using BWA mem and merged and de-duplicated using Picard. Re-alignment around known and novel indel-sites was performed with GATK. All SAM/BAM-conversion steps were completed using SAMtools. Germline samples (T-lymphocytes or PBMC) were compared to reference genome GRCh37 using GATK. However, as the PBMC samples also included CLL cells, no distinction could be made between somatic mutations or novel germline variants for these patients; instead, the PBMC samples of patients 1B and 2B were used to confirm if germline variants identified in sibling(s) were shared. Somatic variation in CLL clone *vs* germline was annotated by the Strelka2 Small Variant Caller. The Variant Call Format (VCF) files were filtered for PASS variants, annotated with VEP and converted to Mutation Annotation Format (MAF) files using VCF2MAF. MAF-files were analyzed using the maftools R package (38). Somatic mutations in CLL-associated genes were annotated by the Ensembl Variant Effect Predictor (VEP, ensembl.org/info/docs/tools/vep/index.html). The panel of CLL driver genes was based on landmark WGS and WES studies (4, 12), for the full panel see **Supplementary Table 1**. Additional screening was then performed for genes in KEGG pathways related to DNA replication, DNA repair, BcR, p53 signaling, cell cycle and the spliceosome. Germline variants were filtered based on clinical significance in ClinVar (https://www.ncbi.nlm.nih.gov/clinvar/), allele frequency, SIFT, PolyPhen and CADD score. All somatic mutations were screened for disease recurrence in CLL and cancer in COSMIC (39) (cancer.sanger.ac.uk) and Intogen (www.intogen.org). The WGS dataset and immunogenetic sequencing data are available upon request to the corresponding author through the SciLifeLab repository (DOI: 10.17044/scilifelab.14932062).

## Results

### Families With Multiple CLL Patients

In family 1 (**Figure 1A**) four (out of a total ten) siblings, i.e. two brothers and two sisters, suffered from CLL. They were diagnosed at advanced age [85 (1A), 86 (1B), 79 (1C), and 60 (1D)] and were followed until late age (98, 91, 84 and 82 years, respectively) (40). All ten siblings grew up on a Dutch farm, where cattle breeding and agriculture were practiced. No record was kept of pesticide use at the farm. All of the other six siblings had passed away at time of inclusion, without showing clinical signs of hematological or immunological disease. Both male patients (1A and 1B) moved out during adolescence, while the female patients 1C and 1D remained at the farm until they were middle-aged. Only patient 1A, who also presented with lymphadenopathy, received treatment for CLL (chlorambucil), twelve years after diagnosis (40). The two brothers of family 2 (**Figure 1B**) were diagnosed with CLL at age 77 (2A) and 71 (2B) years. Family 3 also consisted of two brothers and two sisters with CLL, who were diagnosed in the age range from 64 until 81 years (**Figure 1C**). Sibling 3B was treated with fludarabine. Members of both family 2 and family 3 had the Dutch nationality and were Caucasian. Additional clinical data and descriptive information were unfortunately not available for families 2 and 3.

### Familial CLL Shows Consistent BcR IG Characteristics

Through IG Sanger sequencing of genomic DNA from total PBMC fractions from CLL patients in each of the three CLL families, we discovered strikingly similar immunogenetic features within each family (**Figure 1**). For family 1, a consistent somatic hypermutation (SHM) status of the IGHV gene was observed, with each of the four siblings harboring a M-CLL clone with an IGHV gene germline identity below 98% (**Table 1**). Moreover, two siblings, 1B and 1D had multiple CLL clones, each of which expressed a mutated IGHV gene. Although CLL is generally of monoclonal origin, multiple productive IGH rearrangements have been observed in around 2% of CLL cases (16). These can arise from a single CLL clone (biallelic rearrangement) or reflect biclonal CLL disease (41, 42). Interestingly, family member 1D appeared to have biclonal CLL consisting of a SmIgκ+ and a SmIgλ+ CLL clone as determined by flow cytometry (data not shown). Since we detected three productive IGH rearrangements, one of the two CLL clones likely expresses two IGH alleles. The multiple productive IGH rearrangements identified for family member 1B may also be biallelic but could not be discerned as only one rearranged Ig light chain gene was expressed. Previously, multiple additional IGH bands were detected for these family members in Southern blot analysis (40), but these were now all found to be unproductive. Altogether, family 1 is characterized by M-CLL, with multiple productive and unproductive rearrangements in two individuals.

**Table 1:** Overview of BcR IG sequencing results.

Family member	Stereotyped subset	IGHV gene	HCDR3	IGLV/IGKV gene	LCDR3
**1A**	–	V1-3	CARGVRFLEFLLYGDDAFDIW	IGKV1-33 IGK1-9	CQQYDNLPPALATVCQQVNSYPRITF
**1B**	–	V4-34 V3-15	CARSLVVPAAYGPNSWFDSW CATGGHCGGACYSPYFDYW	IGLV2-18	CSLYTGTKTIF
**1C**	–	V3-7	CAKHDNTGDFHLDNW	IGKV1-16 IGLV2-11	CQQYNSYPALTF CCSYAGSHTYVF
**1D**	–	V1-8 V2-5 V3-15	CARHPSRRCSGDFCSTGNWFDPW CLGHWVRGIMTPFDYW CNYYVMDVW	IGKV3-20 IGLV2-14	CQQYGSSPNTF CSSYTSSNTLVF
**2A**	–	V4-31	CARLLAGLHYYYYYAMDVW	IGKV1-33	CQQYDNLPPYTF
**2B**	–	V4-34	CARERRDSNYGSGIFYYYYGMDVW	IGKV4-1	CQQYYSTPRTF
**3A**	IGLV3-21^R110^	V1-46	CARAWSSAWKYYFDY	IGLV3-21	CQVWDSGSDHPWVF
**3B**	IGLV3-21^R110^/#169	V3-48	CARDGVGAPY	IGLV3-21	CQVWDSGTDHPWVF
**3C**	IGLV3-21^R110^/#2	V3-21	CARDQNGMDV	IGLV3-21	CQVWDSSSDHPWVF
**3D**	IGLV3-21^R110^	V3-48	CARDGGPCGDCYQ	IGLV3-21	CLVWDSGSDHPYVF

In family 2, both siblings expressed unmutated IGHV genes (U-CLL). Notably, each sibling expressed IGHV4 (IGHV4-31 or IGHV4-34), IGHJ6 and IGK light chain genes, but no BcR IG stereotypy was observed (**Table 1**). Hence, the key defining feature of family 2 is the U-CLL type.

Finally, the CLL clone of all siblings of family 3 expressed an IGHV gene with (near) borderline IGHV mutational status (germline identity ranging from 96.4 - 98.2%; borderline IGHV mutational status is classically defined as 97-97.9% germline identity) (43). Notably, the CLL clone in all four siblings utilized the lambda IGLV3-21*04 gene, suggestive of membership of the recently discovered IGLV3-21^R110^ subset (20, 21), which usually has a borderline mutation status. As the BIOMED-2 IGLV/IGLJ light chain consensus primers did not capture the essential final nucleotide of the IGLJ gene to verify the R110 status, we repeated sequencing with adapted primers on cDNA in cases where RNA was available. We confirmed the somatic R110 mutation and germline configuration of the K16 and YDSD motifs in all four members of family 3 (**Supplementary Figure 1**). Regarding the heavy chain, two family members (3B and 3C) belonged to the closely related and clinically aggressive subsets #2 and #169, respectively (44). The respective IGHV genes of these heavy chain stereotypic CLL subsets, IGHV3-21 and IGHV3-48, were 97% identical. The CLL clone of sibling 3B also expressed the IGHV3-48 gene, though the variable heavy CDR3 (VH CDR3) of this patient did not match a stereotyped subset (**Table 1**). Thus, family 3 is paradigmatic for the IGLV3-21^R110^ subset with a borderline IGHV mutation status.

### CLL Families Show Similar Genomic Profiles

To further explore the genomic profiles in these immunogenetically paradigmatic families, we utilized SNP array analysis. We detected genomic aberrations in all three families (**Table 2**). For family 1 and 3 we observed the most common deletion in CLL, del(13q), in the CLL clone(s) of all members, whereas the two brothers of family 2 carried trisomy 12. Additionally, sibling 3C carried del(11q), which is in line with previous reports of subset #2 patients having an increased incidence of 11q deletions (45). Lastly, sibling 1C carried a 2q34-2q35 deletion, a chromosomal aberration not previously associated with CLL, though deletions of 2q37 encompassing *SP140* and *SP110* have been reported (12, 46). Furthermore, the SNP array revealed a distinct loss of heterozygosity (LOH) profile for each family, composed of loci previously linked to CLL in GWAS (**Supplementary Table 3**) (26, 29–33). All three families shared LOH in the MHC locus (6p22.1) and the *CASP8* and *CASP10* locus (2q33.1). LOH of chromosome region 11q22.3, where the *ATM* gene is located, was detected in members of family 1. Additionally, we observed LOH of 14q32.2-q32.33 in family 2, which is interesting as 10% of CLL patients with trisomy 12 were previously observed to have an additional translocation in 14q32 (11). Furthermore, we observed LOH in the 2q22.1 locus in family 2 and family 3, which was recently identified as a novel CLL risk locus using shared genomic segment analysis and was found to include the full *CXCR4* gene. Although there were no cytogenetic data available, we have used SNP array data to define genomic complexity. In none of the patients a high genomic complexity was indicated (**Table 2**), defined as 5 or more unbalanced aberrations according to Leeksma et al. (47–49). Only two patients (1C and 3C) presented with three or four aberrations (**Supplementary Table 4**). In conclusion, SNP-array analysis revealed shared CLL-associated chromosomal aberrations within each family and LOH in several CLL risk loci, and no complex karyotype cases in any of the families.

**Table 2:** Cytogenetic aberrations encountered for each of the three families.

Family member	del(13q)	+12	del(11q)	del(17p)	del(2)(q34q35)	total abberations*
Sibling 1A	yes	no	no	no	no	2
Sibling 1B	yes	no	no	no	no	1
Sibling 1C	yes	no	no	no	yes	4
Sibling 1D	yes	no	no	no	no	1
Sibling 2A	no	yes	no	no	no	1
Sibling 2B	no	yes	no	no	no	1
Sibling 3A	yes	no	no	no	no	1
Sibling 3B	yes	no	no	no	no	1
Sibling 3C	yes	no	yes	no	no	3
Sibling 3D	yes	no	no	no	no	1

*Total number of aberrations >5 Mb, including the recurrent FISH aberrations shown here.

### Whole-Genome Sequencing Identifies Germline Variants in CLL-Related Pathways in All Three Families

**Figure 2:**
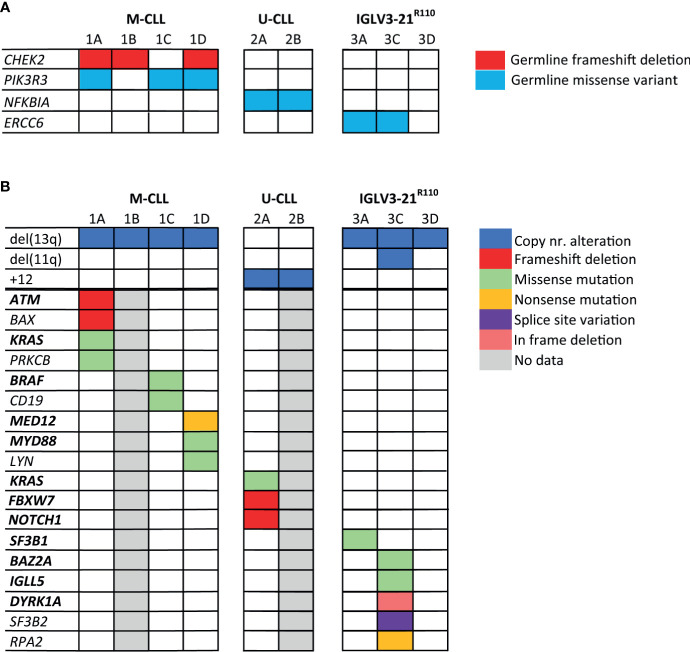
Somatic mutations and germline variants detected by whole genome sequencing of the CLL families. In this figure, both germline variants **(A)** and somatic genetic alterations **(B)** detected in the CLL families are shown. The genes highlighted in bold text are genes, which have previously been identified as CLL driver genes. Genes that are not in bold text were identified during KEGG pathway analysis. Only mutations/variants with likely functional consequences related to CLL development are shown; mutations/variants that were previously reported to be benign or evaluated as benign by variant effect predictors were not shown.

To investigate somatic mutations in the CLL clones and review potential contributing germline variants, we performed WGS on both sorted CLL samples and normal T cells of all three families. Unfortunately, for patients 1B, 2B and 3B, sufficient CLL-derived genomic material for WGS was not available (**Supplementary Table 3**). However, for patient 1B and 2B, we were able to sequence leftover DNA from unsorted PBMCs, allowing us to screen for potential shared germline variants that were found in the families or their members. We performed an initial screen for germline variants and somatic mutations and in CLL driver genes previously identified in WGS and WES studies and then followed up with KEGG pathway analysis to screen for novel CLL-related genes (**Figure 2** and **Supplementary Table 5**).

First, we catalogued the germline variants in each of the families. We identified a germline frameshift deletion in *CHEK2* (p.T410Mfs*15) in siblings 1A, 1B and 1D, but not in sibling 1C (**Figure 2A** and **Supplementary Table 5**). Deleterious germline CHEK2 variants have been associated with an increased risk of developing primarily breast cancer and colorectal cancer (50). Moreover, somatic *CHEK2* alterations have been reported in CLL (4, 51). Additionally, we identified a rare germline missense variant (p.R325C) in *PIK3R3* in sibling 1A, 1C and 1D, but not in sibling 1B. *PIK3R3* is a regulatory subunit of phosphatidylinositol 3-kinase (PI3K) and thus an essential part of the *PI3K/AKT* signaling pathway involved in cell survival and proliferation (52–54).

Notably, we observed a rare germline missense variant in *NFKBIA* (p.T185M) in both siblings of family 2, predicted to be pathogenic by variant effect predictor tools. *NFKBIA* inhibits NF-κB/REL complexes during inflammatory response. *NFKBIA* is also a part of the BcR signaling pathway (55). In family 3, sibling 3A and 3C carried a germline missense variant in the *ERCC6* gene (p.R666C), which encodes a protein involved in the base excision repair pathway. Altogether, some interesting germline variants were observed, but many were of unknown significance and most variants were not shared by all siblings with CLL, making a strong causal relationship in familial CLL less straightforward than for previously described somatic mutations in CLL.

### Known and Novel Somatic Mutations in CLL-Driver Genes and Related Pathways in All Three Families

Next, we characterized somatic mutations specific to the CLL clone (**Figure 2B**). We encountered a somatic frameshift deletion and missense mutation in *ATM* in sibling 1A, which in combination with the LOH of chromosome region 11q22.3 results in bi-allelic loss of *ATM*. This same clone had an additional p.G13D somatic missense *KRAS* mutation and a somatic frameshift deletion in the *BAX* gene. Lastly, we observed a somatic missense mutation (p.D470H) in sibling 1A in the *PRKCB* gene, involved in many different signaling pathways, including B-cell activation.

The CLL clone of sibling 1C carried two somatic missense mutations of interest, a p.D594N mutation in *BRAF* previously observed in CLL, and a novel *CD19* mutation (p.L495P). Sibling 1D presented with biclonal CLL, one SmIgκ^+^ and one SmIgλ^+^ CLL clone. In each CLL clone, a known CLL driver gene was affected; the IGK+ clone carried a truncating mutation in *MED12* (56), while the IGL^+^ clone carried a missense mutation at the CLL hotspot (L273P) in *MYD88* (57). Furthermore, we observed a somatic missense mutation (p.G2R) in both the SmIgκ^+^ and SmIgλ^+^ clones in *LYN*, a gene directly downstream of the BcR.

In family member 2A we detected somatic frameshift deletions in *FBXW7* and *NOTCH1* and a missense mutation in *KRAS*, all of which have been previously associated with the occurrence of trisomy 12 in CLL (11, 58–60). Unfortunately, the lack of somatic data from patient 2B prevented us from confirming if the somatic mutational profile matched between siblings.

In family 3, we observed a somatic mutation in one of the CLL hotspots (p.G742D) of *SF3B1* for sibling 3A. *SF3B1* mutations are common in CLL and particularly associated with subset #2 and the IGLV3-21^R110^ subset (20, 21). Sibling 3C carried somatic mutations in several low-frequency mutated genes in CLL: *IGLL5*, *DYRK1A* and *BAZ2A* (4, 12). The somatic mutation in *IGLL5* is likely the result of aberrant SHM (61). Additionally, sibling 3C carried a somatic truncating mutation in *RPA2*, a gene involved in DNA replication and repair. In contrast, no noteworthy somatic mutations were observed in sibling 3D.

In summary, the WGS results yielded several somatic mutations in recurrently mutated genes in CLL, as well as four germline variants in genes in CLL-associated pathways, though there was limited overlap in the genes affected by the somatic mutations in members within and across families.

## Discussion

In this study, we describe three families that represent distinct immunogenetic subgroups of CLL, presenting a unique opportunity to study the contribution of genetics and immunogenetics in CLL pathobiology. Each of the three families developed CLL with a consistent IGHV SHM status, encompassing one of three prototypes of the IGHV SHM spectrum: i.e. U-CLL, M-CLL and borderline mutated CLL. While families 1 and 2 reflect the M-CLL and U-CLL subgroups, respectively, family 3 presented with borderline mutated CLL and all family members carried the lambda IGLV3-21 light chain. Furthermore, family 3 expressed the IGLV3-21*04 allele and displayed the R110 mutation characteristic of the IGLV3-21^R110^ subset. This light chain was paired with a stereotyped VH CDR3 of the immunogenetically related subsets #2 and #169, both of which belong to the broader IGLV3-21^R110^ category. We observed distinct profiles of genetic alterations for each of these families, with further unique somatic mutations for each sibling. While our results are consistent with previous associations between IGHV SHM mutational status and specific genetic aberrations in CLL driver genes, the similarities in (immuno)genetic features within each family highlight their important contribution to the onset and evolution of familial CLL.

The dichotomy between U-CLL and M-CLL is thought to originate from the B-cell maturation process after antigen activation (62). For M-CLL, the antigen-activated B cell follows the traditional path of T cell-dependent germinal center B cell maturation. For U-CLL, the antigen-activated B cell is thought to mature largely independent of the T cell influence (62). Throughout these processes, chronic antigenic stimulation through (auto)antigens would keep the B cell in a constant state of activation. For the IGLV3-21^R110^ subset, this constant activation is most probably the result of autostimulation through BcR aggregates on the cell surface.

As would have been expected based on the association of IGLV3-21 with CLL with limited SHM activity, the IGLV3-21^R110^ subset is characterized by a (near) borderline mutational status (63). Correspondingly, no cases of IGLV3-21^R110^ with 100% IGHV germline identity have been encountered, thus supporting SHM as the mechanism for the introduction of the somatic R110 mutation (20). The IGHV germline identities of the IGLV3-21^R110^ CLL family 3 follow a similar pattern, ranging from a germline identity of 96.4% to 98.2%. Interestingly, usage of the IGLV3-21*01 or *04 alleles gives an inherent risk of IGLV3-21^R110^-related CLL, due to the germline presence of the K16 and YDSD motifs (20). Our findings in the current study would support the theory that this inherent risk contributes to the increased incidence of CLL among relatives of CLL patients.

We additionally observed a somatic mutation in *SF3B1* for sibling 3A. *SF3B1* mutations are common in CLL and particularly associated within the IGLV3-21^R110^ subset (20, 21). As the SF3B1 protein is a component of the spliceosome, we screened for additional mutations in the spliceosome pathway. We discovered that sibling 3C carried a splice site alteration in *SF3B2*. Unlike *SF3B1*, *SF3B2* has never been independently linked to CLL. The finding of a splice site alteration in *SF3B2* in sibling 3C suggests that the alterations in other genes involved in the spliceosome may be relevant for the IGLV3-21^R110^ subset as well, although this awaits further confirmation in larger cohorts.

We identified several germline variants of unknown significance (VUS) in each of the families by KEGG pathway analysis. Family 1 presented with germline variants in *CHEK2* and *PI3KR3*, while family 2 carried a germline variant in *NFKBIA* and two siblings of family 3 carried a germline variant in *ERCC6*. *CHEK2* is a gene associated with DNA damage and repair as well as cell cycle regulation and apoptosis in response to DNA damage (51). Somatic *CHEK2* mutations have been identified as putative CLL drivers, while *CHEK2* germline variants have recently been indicated as a novel predisposition gene in CLL, implying that CLL may belong to the spectrum of malignancies associated with germline variant in CHEK2 (54). In addition, three out of four siblings with CLL carried a rare germline variant in *PIK3R3*, an essential component of the PI3K/AKT signaling pathway. Recently, altered activation of the PI3K/AKT signaling-pathway was identified as a critical component of sustained proliferation and survival in CLL (64). During this process, autonomous autoreactive BcR signaling typically converges with activation of the PI3K/AKT signaling-pathway (64). While germline variants in components of the PI3K/AKT pathway could theoretically contribute to this aspect of CLL development, no convincing supporting evidence for a role of any germline variant has this far been reported. NFKBIA is part of the NF-κB and BcR signaling pathways and its expression has been suggested as a biomarker for risk stratification in DLBCL (55). *ERCC6* has a role in base excision repair, particularly during transcription.

Our study was limited by its sample size as well as by the amount of material available for each patient. Additionally, clinical follow up data was not available for family 2 and 3 and no material was available from healthy family members. Lastly, the absence of conventional chromosomal analysis may have affected the identification of complex rearrangements (>3 abnormalities), a prognostic factor in CLL, although we feel that based on SNP array data we could exclude the occurrence of complex karyotype cases. Nevertheless, we feel that the three families are paradigmatic for the main CLL subgroups and as such provide a platform for further studies into the link between immunogenetics and genetic predisposition. That said, environmental factors like pesticides, herbicides and pathogens could be relevant risk factors in familial CLL as well. This would especially apply to the siblings of family 1, who all grew up on the same farm (65). Unfortunately, as no toxicological or biological measurements were done, the contribution of these factors to CLL development in family 1 remains unclear.

In summary, we evaluated immunogenetic, cytogenetic, germline and somatic lesions in familial CLL. In each family, a consistency of IGHV mutational status was observed, with the particularly intriguing finding that all individuals in one of the families belonged to the IGLV3-21^R110^ CLL subset. Furthermore, we highlight the co-occurrence of specific genetic aberrations and germline variants within each family, pointing towards shared underlying mechanisms in CLL development. Our data warrants a more comprehensive evaluation of this potential association between germline predisposition and immunogenetic features in the development of CLL.

## Data Availability Statement

The datasets presented in this study can be found in online repositories. The names of the repository/repositories and accession number(s) can be found below: https://figshare.com/s/f8576aca6650fab99393, 10.17044/scilifelab.14932062.

## Ethics Statement

The studies involving human participants were reviewed and approved by Erasmus MC Medical Ethics Review Committee. The patients/participants provided their written informed consent to participate in this study. Written informed consent was obtained from the individual(s) for the publication of any potentially identifiable images or data included in this article.

## Author Contributions

AM and IW-T performed the experiments. PK, AM, VL, KP, and ND analyzed the data. AA validated CLL subsets. PK, AM, VL, RR, and AL interpreted results. PK and AL wrote the manuscript. AM, VL, AA, PH, KP, ND, HB, RH, JD, and RR critically reviewed and edited the manuscript. AL designed and supervised the study. All authors contributed to the article and approved the submitted version.

## Funding

The SNP&SEQ Platform is also supported by the Swedish Research Council and the Knut and Alice Wallenberg Foundation. RR is supported by SciLifeLab, the Swedish Cancer Society, the Swedish Research Council, the Knut and Alice Wallenberg Foundation, Karolinska Institutet, Karolinska University Hospital, and Radiumhemmets Forskningsfonder, Stockholm. PK and AL are supported by a EU TRANSCAN-2/Dutch Cancer Society grant (179;NOVEL consortium).

## Conflict of Interest

The authors declare that the research was conducted in the absence of any commercial or financial relationships that could be construed as a potential conflict of interest.

## Publisher’s Note

All claims expressed in this article are solely those of the authors and do not necessarily represent those of their affiliated organizations, or those of the publisher, the editors and the reviewers. Any product that may be evaluated in this article, or claim that may be made by its manufacturer, is not guaranteed or endorsed by the publisher.

## Supplementary Material

Supplementary Figure 1 Validation of the IGLV3-21*04^R110^ mutation in family 3. Identification of the IGL rearrangement in WGS data of sibling 3C by the ARResT/Interrogate immunoprofiler (upper segment) and a complimentary view in Integrated Genome Viewer (IGV, lower segment) highlighting the g>c missense mutation at the last base in IGLJ3 resulting in the G110R substitution.

Supplementary Table 1 Overview of CLL driver genes and KEGG pathways used to analyze the WGS data.

Supplementary Table 2 Genomic profile for CLL loci for each family

Supplementary Table 3 Samples utilized for WGS.

Supplementary Table 4 SNP array analysis results with relevant aberrations for complex karyotypes indicated.

Supplementary Table 5 Overview of somatic mutations and germline variants that likely contribute to CLL pathogenesis.
