# Supplementary figures and images for: Consistent B Cell Receptor Immunoglobulin Features Between Siblings in Familial Chronic Lymphocytic Leukemia
Source: Front Oncol. 2021 Aug 26;11:740083. doi: 10.3389/fonc.2021.740083 (PMC8427434; doi:10.3389/fonc.2021.740083)

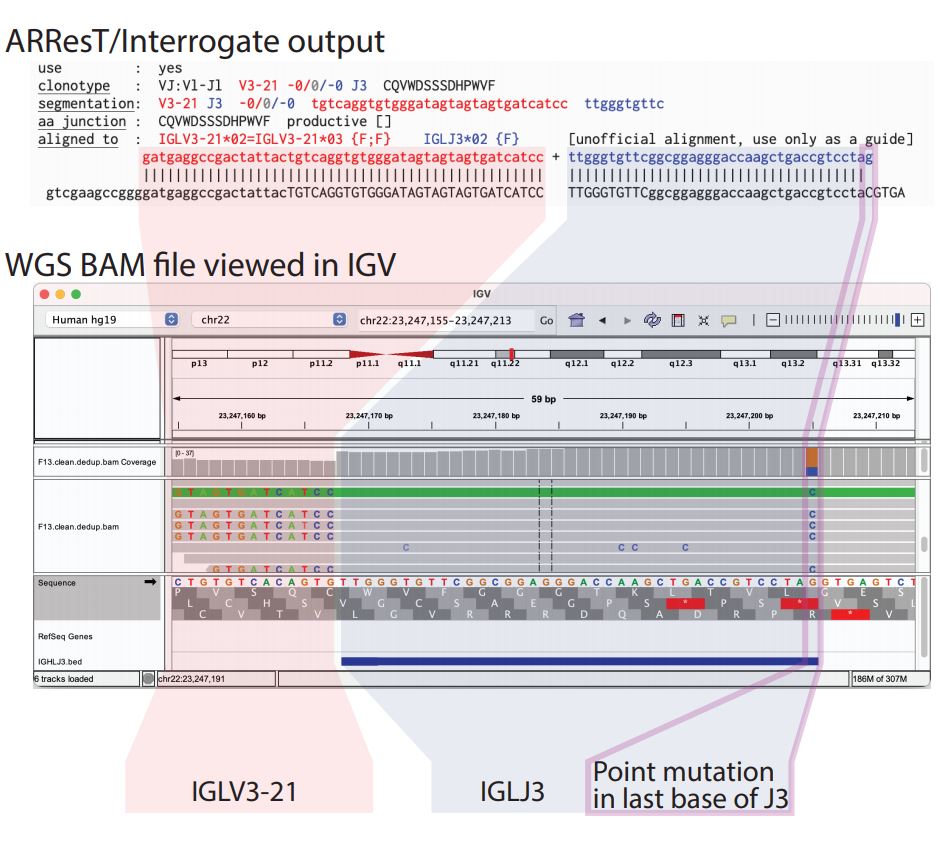

Supplement: Supplementary Figure 1 — Validation of the IGLV3-21*04R110 mutation in family 3. Identification of the IGL rearrangement in WGS data of sibling 3C by the ARResT/Interrogate immunoprofiler (upper segment) and a complimentary view in Integrated Genome Viewer (IGV, lower segment) highlighting the g>c missense mutation at the last base in IGLJ3 resulting in the G110R substitution. [file Image_1.jpeg]
