# Supplementary material for: Consistent B Cell Receptor Immunoglobulin Features Between Siblings in Familial Chronic Lymphocytic Leukemia
Source: Front Oncol. 2021 Aug 26;11:740083. doi: 10.3389/fonc.2021.740083 (PMC8427434; doi:10.3389/fonc.2021.740083)
Supplement: Supplementary Table 4 — SNP array analysis results with relevant aberrations for complex karyotypes indicated. [file Table_4.pdf]

Family 1

Fam member	Aberration type	Cytoband	Size in MB	Genomic localization (nucleotides)	Clone size (%)
**1A**	Loss	6p22.3-6p22.2	1.78	24,469,379-26,246,276	100
	Loss	13q14.2-13q14.3	1.49	50,065,667-51,551,320	90
**1B**	Loss	13q14.2-13q14.3	2.81	49,523,937-52,338,243	90
**1C**	Loss	2q34-2q37.1	20.3	211,514,492-231,825,834	15
	CNLOH	6p25.1-6p21.33	25.4	6,041,659-31,485,230	15
	Loss	13q14.2-13q14.3	0.858	50,510,401-51,368,308	50
	Loss	17pter-17p11.2	17.5	1-17,583,834	15
**1D_L**	Loss	13q14.2-13q14.3	1.725	49,858,608-51,583,988	95
	Loss	13q14.2-13q14.3	0.892	50,554,402-51,447,049	89
**1D_K**	Loss	13q14.11-13q14.3	8.15	44,327,775-52,474,936	90
	Loss	13q14.2-13q14.3	1.725	49,858,608-51,583,988	10

Family 2

Fam member	Aberration type	Cytoband	Size in MB	Genomic localization (nucleotides)	Clone size (%)
**2A**	gain	Chromosome 12	Entire chromosome	Entire chromosome	20
	Loss	14q11.2	0.377	22,577,021-22,954,788	100
**2B**	gain	Chromosome 12	Entire chromosome	Entire chromosome	15
	Loss	14	0.377	22,577,021-22,954,788	100

Family 3

Fam member	Aberration type	Cytoband	Size in MB	Genomic localization (nucleotides)	Clone size (%)
**3A**	Loss	13q14.2-13q14.3	3.17	50,563,447-51,500,576	30
	Loss	17q23	0.745	55,337,081-56,082,191	90
**3B**	Loss	13q14.2-13q14.3	3.17	48,520,428-51,695,552	80
**3C**	gain	8q21.3-8q24.3	59.1	87,166,395-146,285,083	65
	CN-LOH	11q13.2-qter	69,2	65,790,190-135,006,516	65
	loss	13q14.13-13q14.3	4.65	46,794,527-51,447,049	90
**3D**	Loss	1p36.11	0.523	27,067,809-27,591,381	85
	Loss	13q14.2-13q14.3	0.740	50,237,742-50,977,946	80
